# Proteomics analysis of faecal proteins in the tick *Haemaphysalis flava*

**DOI:** 10.1186/s13071-018-2673-3

**Published:** 2018-02-08

**Authors:** Lei Liu, Yi-song Liu, Guo-Hua Liu, Tian-yin Cheng

**Affiliations:** College of Veterinary Medicine, Hunan Collaborative Innovation Center of Safety Production of Livestock and Poultry, Hunan Agricultural University, Changsha, Hunan Province 410128 People’s Republic of China

**Keywords:** *Haemaphysalis flava*, Tick, Faeces, Proteome, Blood digestion

## Abstract

**Background:**

Ticks and tick-borne diseases are of major public health concern. Currently, development of vaccines against ticks is considered crucial for their control. A critical step in this process is the screening of viable antigens. Faeces are byproducts of digestion and blood meal utilization, and partly reflect the vitality and vector potential of ticks. However, an integrated analysis of proteins in tick faeces is lacking. The present study explored the protein components in the faeces of the tick *Haemaphysalis flava*, by liquid chromatography–tandem mass spectrometry (LC/MS-MS) to identify potential protein antigens for vaccine development against ticks.

**Methods:**

Faeces from adult *H. flava* engorged females were collected. Proteins were extracted from faeces, and the trypsin-digested peptides were analyzed by LC/MS-MS. High confidence proteins were identified based on unique peptides revealed by MS. Potential faecal protein genes, as well as their sources, were also characterized by searching previous transcriptome datasets from the salivary glands and midgut of *H. flava*.

**Results:**

In total, 21 were recognized with confidence. Amongst these, 18 were of likely tick origin, while three proteins (serum albumin, haemoglobin α and β subunits) were likely from hosts. Seventeen unigenes corresponding to these proteins were retrieved by searching our previous *H. flava* salivary glands and midgut transcriptomic datasets. Some proteins were reported to prevent blood clotting, play a role in immunity and antibiosis, and formation of musculature*.* The functions of the remaining proteins are unknown.

**Conclusions:**

Identifying antigens for tick vaccine development is feasible by analyzing the faecal proteome as well as the transcriptomes of salivary glands and midguts. The vast number of proteins detected in tick faeces highlights the complexity of blood digestion in ticks, a field that needs more investigation.

## Background

Ticks (Arthropoda: Arachnoidea) are pests of livestock, pets, wildlife and humans. Infestations result in lesions, emaciation, anaemia, paralysis, and possibly the death of hosts. More importantly, ticks are also reservoirs of pathogens and vectors of a myriad of viruses, bacteria, protozoa, fungi, and helminths [1, 2]. Hence, controlling tick populations will have a significant impact on public health. The current strategy for tick control is to develop vaccines against them [3]. In this regard, screening for potential antigens is a critical step toward effective vaccine development.

Like other arthropods, the digestive tract of a tick consists of foregut, midgut and hindgut. While the hindgut mainly stores undigested wastes, the midgut is a major site for blood meal digestion. Malpighian tubules, which are the excretory organs in ticks, are connected to rectal caeca. Excreta from Malpighian tubules and digestion remnants converge in the hindgut and are discharged into the anal opening. Faeces are byproducts of digestion and blood meal utilization and are also associated with the vitality and vector potential of ticks [4]. Studies on the faecal components of ticks date back to the 1970s. In 1972, Hamdy compared faeces from 10 ticks and identified guanine and an unknown purine compound in these samples [5]. Later, proteins in the faeces of *Hyalomma dromedarii* were reported only during blood-feeding [4]. In 1989, Katsuki et al. [6] discovered that albumins and haemoglobins accounted for 57% to 99% (w/w) of the proteins in the faeces of *Haemaphysalis longicornis* nymphs. Later, Frantisek et al. [7] reported the presence of xanthines and hypoxanthines in the excreta of some argasid tick species. In 2001, Stoyan [8] revealed that excreta of *Ixodes ricinus* contained uric acids and 8-azaguanine, and Daniel [9] detected ammonia in the faeces of *Ixodes scapularis*.

Thus far, integrated analyses of proteins in tick faeces have not been conducted. The present study explored faecal proteins in *H. flava* by liquid chromatography-tandem mass spectrometry (LC/MS-MS) for potential use as protein antigens for vaccine development against ticks.

## Methods

### Tick source and collection of faeces

The flagging method was used to collect ticks in Xinyang, Henan Province (32°13′N, 114°08′E). More than 30 ticks in a non-engorged state were obtained and allowed to feed on hedgehogs in the laboratory. Ticks were harvested after detaching from hosts in a full-engorged state.

Adult female ticks were individually immobilized with their abdomen upwards onto a sterile glass slide using tapes. Each tick was subject to adequate stimuli developed in our laboratory and then kept in a wet box at 30–33°C for 3–4 h. Faeces were collected from the anus and pooled into a clean 0.2 ml centrifuge tube, and mixed with 100 μl of lysis buffer (20 mM Tris-HCl, 0.2% SDS, pH 7.5) and incubated in a boiling water bath for 5 min. Ultrasonic pyrolysis was applied to the mixture for an additional 5 min. Then, the lysates were centrifuged at 15,000× *rpm* for 10 min, and the supernatant was stored at -80 °C until further use.

The conventional bicinchoninic acid (BCA) method was used to evaluate protein levels in the supernatant. An aliquot of the supernatant was subjected to protein analysis using SDS-PAGE.

### Protein preparation for high performance liquid chromatography (HPLC)

An aliquot of the supernatant (50 μg in weight) was mixed with 1,4-dithiothreitol (DTT) to a final concentration of 100 mM, and the mixture was boiled for 5 min and allowed to cool at room temperature. Then, a 200 μl of UA buffer (150 mM Tris-HCl, 8 M urea, pH 8.0) was added to the mixture, vortexed, transferred to an ultrafiltration tube with a 14 kDa membrane, and centrifuged at 14,000× *g* for 15 min. This process allowed proteins to be retained on the membrane, and the supernatant to be discharged. The step was repeated to ensure the complete removal of non-protein impurities. The proteins were then reconstituted in 200 μl of 50 mM iodoacetamide (IAA), shocked at 600× *rpm* for 1 min, and left for 30 min in the dark at room temperature. The remaining liquid was discarded by centrifuging at 14,000× *g* for 10 min, and proteins retained on the membrane were washed twice with 200 μl of UA buffer, and further with 200 μl of dissolution buffer (25 mM NH_4_HCO_3_). The protein extracts were digested with 40 μl of trypsin buffer (3 μg trypsin from Promega in 40 μl dissolution buffer) in a 37 °C water bath for 16–18 h, and the resulting peptides were collected as a filtrate. The peptide concentration of the filtrate was measured at OD280 nm.

### Faecal protein analysis by LC/MS-MS

Peptides were separated using the Easy nLC HPLC system (Thermo Scientific, Waltham, MA, USA). Mobile phase A was 0.1% formic acid, and mobile phase B was 84% acetonitrile (ACN) in 0.1% formic acid. Chromatographic columns were balanced with 95% mobile phase A before sample loading. Samples were injected onto a trap column (2 cm*100 μm 5 μm-C18) by an auto-sampler, and then onto an analytical column (75 μm*100 mm 3 μm-C18). The flow rate was 300 nl/min. Mobile phase B was invoked as an eluent.

Every sample after separation by HPLC was subjected to mass spectrometry analysis using a Q-Exactive^TM^ mass spectrometer (Thermo Scientific). Nanospray ionization (NSI) was used as the ion source, and argon was used as collision gas. The whole analysis time was set at 240 min.

### MS data analysis

Raw data generated by MS/MS was imported into Bruker compass Data Analysis 4.0 (http://bruker-compass-dataanalysis.updatestar.com/). Then, unique peptides were searched in the peptide library conceptually translated from *H. flava* midgut and salivary gland transcriptome datasets (GSE67247, GSE69721, translated by TransDecoder with 25943 sequences in total) and Uniprot database by Mascot 2.0 (Matrix Science, Boston, USA). Carbamidomethylation of Cys was defined as a fixed modification, while oxidation of Met was defined as the variable modification. Searches had tryptic specificity and allowed a maximum of one missed cleavage and tolerance on the mass measurement of 20 ppm in MS mode and 0.5 Da for MS/MS ions. The significance threshold was set at ≥ 95%, and only those proteins with ≥ 2 unique significant peptides were selected.

### Protein identification and database search

Proteins were identified using the peptide library conceptually translated from the *H. flava* midgut and salivary gland transcriptome datasets (https://www.ncbi.nlm.nih.gov/gds/?term=GSE67247; https://www.ncbi.nlm.nih.gov/gds/?term=GSE69721), NCBInr (https://www.ncbi.nlm.nih.gov/protein) and UniProt (http://www.uniprot.org/blast/) databases. UniGenes were also retrieved by searching datasets GSE67247 (https://www.ncbi.nlm.nih.gov/gds/?term=GSE67247) and GSE69721 (https://www.ncbi.nlm.nih.gov/gds/?term=GSE69721), previously deposited in NCBI by our group.

## Results

### Collection of *H. flava* faeces

**Fig. 1:**
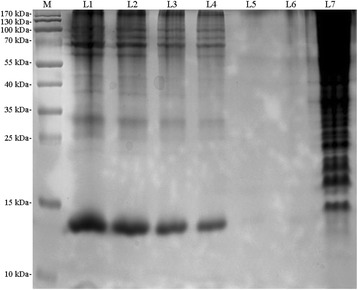
SDS-PAGE analysis of *H. flava* faecal protein extracts. Lane M: marker protein; Lane 1: 18 μl protein extracts; Lane 2: 15 μl protein extracts; Lane 3: 10 μl protein extracts; Lane 4: 7 μl protein extracts; Lane 5: 15 μl blank control (lysis buffer); Lane 6: 10 μl blank control (lysis buffer); Lane 7: positive control

We have developed a feasible method collect tick faeces in the laboratory. Using this method, we collected two batches of faeces from female ticks. Analysis of faecal proteins by HPLC and SDS-PAGE showed the presence of a complex set of proteins in the faecal extracts of *H. flava*, with molecular weights ranging from 15 kDa to 170 kDa (Fig. 1). Robust bands were observed at 13 kDa, 70 kDa, 100 kDa and 170 kDa (Fig. 1).

The protein concentration of faecal extracts was estimated to be 1.39 mg/ml by the BCA method.

### Faecal protein analysis by LC/MS-MS

Overall, we analyzed two batches of faecal proteins by LC/MS-MS and obtained 80 high confidence peptides (≥ 95%) in total. Twenty-one proteins were identified by searching the peptide library conceptually translated from *H. flava* midgut and salivary gland transcriptome datasets, NCBInr and UniProt databases (Table 1). Amongst these, 18 proteins were annotated as tick proteins. However, one protein, serum albumin (A0A140T897), was likely a host protein. The remaining two high confidence proteins, haemoglobin α and β subunits, were likely from hedgehogs, the host used in the present study.

**Table 1:** Proteins with high confidence in *H. flava* faecal protein extracts detected by LC/MS-MS

No.	Unique peptides	Peptides deduced from	Coverage (%)
1	AGFAGDDAPR; CDVDIR; DSYVGDEAQSK; IIAPPER; LCYVALDFEQEMATAASSSSLEK; QEYDESGPSIVHR; VAPEEHPVLLTEAPLNPK	cds.Contig217 in GSE67247	23.2
2	AAEQSLEESQARVNELTTINVNIAAAKNK; EKSDLTVQLMQLSDR; RQLNEQEGLSQQNLTR; YQAQITELEMSLDAANKQNMDLQK	cds.Contig2051 in GSE69721	9.6
		cds.Contig27282 in GSE67247	9.6
3	CLTDIQAGLEK; ELIGFVAEGSQELFK; HAPCLGQTLPDQK; HAPCLGQTLPDQKK	cds.Contig2959 in GSE67247	17.2
4	DANECLPQEVAGCD; VADPDDCGK; VNCPPLQHFSK; YSLCTATFSTK	cds.Contig6112 in GSE67247	36.3
5	IQIVGDDLTVTNPK; LAVQEFMILPTGATTFTEAMK; YMGKSVFKAVDNINK	cds.Contig2263 in GSE67247	11.3
6	YVPDPDDCTK; YTVCSGGFGMK	cds.Contig13170 in GSE67247	38.9
7	VEGLENYDTVLELTK; VETQVVAGLNYR	cds.Contig499 in GSE69721	24.8
8	EIQTAVR; LLLPGELAK	cds.Contig27398 in GSE67247	11.0
9	CPCYGTDR; GCASATSVLTR	cds.Contig6678 in GSE69721	24.4
		cds.Contig27530 in GSE67247	11.4
11	VDKLMGR; VTDGALVVVDCVSGVCVQTETVLR	cds.Contig198 in GSE69721	3.7
10	DQAGEFNTR; TDDLCAVQK	cds.Contig3975 in GSE69721	8.9
12	SVDFANEGPR; YNLVPAMR	cds.Contig7475 in GSE67247	10.3
		cds.Contig36225 in GSE69721	15.1
13	SVGSFEFQSTLPADASPK; FTEAEITTEQTDR	cds.Contig4707 in GSE67247	6.6
14	AAPEVGDGAAK; LDNGVIAPFDPYLDLK	cds.Contig15192 in GSE69721	21.1
15	ALVTGLWGK; DFTPAAQAAFQK; FFDSFGDLSSADAVMGNPK; VKVEEFGGEALGR; VLQSMGDGIK; VHLTAEEK; LSELHCDK; LHVDPENFR; NLDNLK	P02059 in UniProt; Hemoglobin subunit β; *Erinaceus europaeus*	67.4
16	LGGHGGEYGGEALDR; LRVDPVNFK; MFQAHPTTK; VDPVNFK; FQAHPTTK	P01949 in UniProt; Hemoglobin subunit α; *E. europaeus*	23.4
17	AEFVEVTK; GACLLPK; HLVDEPQNLIK; KVPQVSTPTLVEVSR; LVTDLTK; LVVSTQTALA; QNCDQFEK; QTALVELLK; YICDNQDTISSK	A0A140T897 in UniProt; Serum albumin; *Bos taurus*	18.5
18	ICELSSDTHQEHANTFLPDDVK; LNCEDLDCVFTK; QRLNCEDLDCVFTK	A1Y1T6 in UniProt; Neutrophil elastase inhibitor; *Rhipicephalus microplus*	27.7
19	CPEATNYGFLIFAR; FEAEDNGTPCQTK	G3MTW3 in UniProt; Uncharacterized protein; *Amblyomma maculatum*	17.7
20	HVPDYCTFVFNVFCAK; HVPDYCTFVFNVFCAKDR	Q8WSK7 in UniProt; Serotonin and histamine binding protein; *Dermacentor reticulatus*	16.8
21	ANIPRWYYDTNNATCEMFTYGGITGnKNNFESEEECK; CNESCNDAPKPPCSLEVDYGVGR	Q964Q0 in UniProt; Ixolaris; *Ixodes scapularis*	36.4

Based on protein annotations, we searched the *H. flava* salivary gland (GSE67247) and midgut (GSE69721) transcriptomic libraries and selected 17 unigenes encoding these proteins (Table 2). Following alignment of proteins with other tick species, 13 unigenes were found to be homologs of actin, enolase, mucin, AV422, elongation factor 2, cysteine-rich protein, histone H2B, serpin, paramyosin and microplusin-1. However, Contig13170, Contig4707, Contig15192 and Contig-499 were found to encode a fraction of hypertrophic, α-2-macroglobulin, chitinase and cystatin, respectively. The identities between the *H. flava* proteins and their tick homologues were highly variable, and ranged from 41% to 100%. Notably, *H. flava* enolase had an 84% amino acid (aa) identity to the *I. ricinus* homologue, and an 82.4% aa identity to the *Ornithodoros moubata* homologue; AV422 in *H. flava* showed 95.2% identity with the *Amblyomma americanum* homologue; *H. flava* histone H2B had 98.4% of identity with the *I. scapularis* homologue; the identity of *H. flava* paramyosin was 98.1%, 97% and 94% compared with the *H. longicornis*, *Rhipicephalus microplus* and *I. scapularis* homologues, respectively; homology of the elongation factor-2 in *H. flava* was 98.3%, 97.7%, 97.7%, 97.7%, 96.3% and 90.5% compared with *Rhipicephalus appendiculatus*, *Amblyomma parvum*, *Amblyomma cajennense*, *Amblyomma aureolatum*, *Hyalomma excavatum* and *I. ricinus*, respectively. However, unigenes corresponding to ixolaris, neutrophil elastase inhibitor, serotonin and histamine binding protein were not identified.

**Table 2:** Unigenes from transcriptome datasets homologous to the proteins identified by faecal proteomic analysis

No.	Unigene		Putative protein				
	ID	Nucleotide length (bp)	Protein length (aa)	Protein annotation	E-value	Score	Identity (%)
1	Contig217 in GSE67247	1521	376	Q6X4V4, actin, *R. microplus*	0.0	1967	100.0
2	Contig2051 in GSE69721	3161	877	J7LVN2, paramyosin, *H. longicornis*	0.0	4180	98.1
	Contig27282 in GSE67247	3907	877				
3	Contig2959 in GSE67247	1211	231	M4PPE7, AV422, *A. americanum*	9.3e-164	1186	95.2
4	Contig2263 in GSE67247	2372	433	D4P967, enolase, *Ornithodoros moubata*	0.0	1878	82.4
5	Contig6112 in GSE67247	444	124	Q2PGH6, mucin, *H. longicornis*	1.2e-59	487	74.5
6	Contig13170 in GSE67247	787	230	A0A023G8D0, pertrophin, *A. triste*	7.9e-36	301	90.7
7	Contig499 in GSE69721	467	109	A0A023G8F8, cystatin-2, *A. triste*	8.8e-35	310	80.3
8	Contig27398 in GSE67247	784	125	A0A131Y6E4, histone H_2_B, *I. scapularis*	1.2e-79	614	98.4
9	Contig6678 in GSE69721	398	78	A0A034WXB2, cysteine-rich protein, *R. microplus*	4.4e-22	224	65.8
	Contig27530 in GSE67247	751	167		3.3e-31	301	54.1
10	Contig198 in GSE69721	2899	847	A0A131Z5S6, elongation factor 2, *R. appendiculatus*	0.0	4329	98.3
11	Contig3975 in GSE69721	1669	202	A0A023FTF7, microplusin-1, *A. maculatum*	2.3e-71	567	72.8
12	Contig7475 in GSE67247	937	174	Q75Q63, serpin-2, *H. longicornis*	3e-108	295	90.0
	Contig36225 in GSE69721	359	119		1e-61	202	90.0
13	Contig4707 in SE67247	1665	551	A0A023FLU0, α-macroglobulin, *A. cajennense*	0.0	2323	80.4
14	Contig15192 in SE69721	494	128	A0A02323FPH1, chitinase, *A. cajennense*	9.7e-54	468	73.8

## Discussion

We have identified 21 proteins in the faeces of the tick, *H. flava.* Amongst these, 18 were confirmed to be of tick origin based on the retrieval of their protein homologs by searching databases of *H. flava* salivary gland and midgut transcriptomes. To our knowledge, this is the first faecal proteomic study in ticks.

The life-cycle of hard ticks includes four developmental stages, i.e. eggs, larvae, nymphs and adults. Except for eggs, ticks of the last three stages require blood-feeding for their survival. It is likely that serum albumins may have originated from hosts of *H. flava* nymphs because these do not digest serum albumins completely, as reported for *H. longicornis* nymphs [6]. Haemoglobin α and β subunits from hedgehogs were also detected in the faeces of *H. flava*. This observation was consistent with other reports of haemoglobin in ticks faeces based on spectrophotometry [4, 6]. Furthermore, our search for homologs of ixolaris, a neutrophil elastase inhibitor, serotonin and histamine binding proteins and an uncharacterized protein did not yield any positive result. The functions of five proteins, namely cysteine-rich protein, mucin, elongation factor-2, hypertrophic and microplusin-1, are unknown. The remaining 11 proteins may function in prevention of blood clotting, immune-mediation and antibiosis, and formation of musculature in ticks.

Enolase, AV422, serpin-2 and cystatin-2 are proteins that hinder blood clotting, but their underlying mechanisms of action vary. Xu et al*.* [10] revealed the full-length enolase gene to be 1988 bp with an open reading frame (ORF) containing 1302 bp that encodes a protein with 433 aa. Recombinant enolase could bind human plasminogen, a key clotting factor, which could be activated during coagulation in a dose-dependent manner. An enolase from *Ornithodoros moubata* is secreted into the saliva, where it functions as the receptor of plasminogen to stimulate fibrinolysis in hosts, in order to maintain the fluidity of the blood during feeding [11]. Further, RNAi and immunization studies have demonstrated that inactivating enolase could affect tick reproduction, indicating that this could be a new strategy for tick control [11].

The full-length AV422 gene in *H. flava* was 1152 bp, encoding a protein with 231 aa (unpublished data). Recombinant AV222 could significantly extend the prothrombin time (PT), thrombin time (TT) and activated partial thromboplastin time (APTT) *in vitro* (unpublished data). AV422 in *Amblyomma americanum* has been reported to mediate anti-haemostasis and anti-complement processes during feeding by postponing the plasma clotting time, preventing platelet aggregation, and reducing the final complement complexes [12]. It was suggested that AV222 could be a potential candidate antigen for vaccine development against ticks, consistent with our observation.

The full-length serpin-2 gene in *H. flava* was 1467 bp, encoding a protein of 398 aa with a signal peptide of 17 aa (unpublished data). The protein structure and function of *H. flava* sepin-2 was similar to serpin-2 in *H. longicornis*, but not to serpin-1 [13]. They shared 87% aa identity, and both could significantly delay APTT.

Moreover, Contig499 identified in the transcriptomic library of midguts encoded a protein with 78% aa identity with Hlcyst-3, a member of cystatins family. Over the last two decades, several cystatins from different tick species have been identified, and their biochemical functions have been analyzed concerning the physiology and blood-feeding lifestyle of ticks. Zhou et al. [14] confirmed that recombinant Hlcyst-3 could inhibit papain and cathepsin L and that its expression was highest in tick midguts.

The full-length sequence of histone H2B was 124 aa based on two unigenes, Contig27398 and Contig1248. It could react with rickettsial adhesin OmpB, thus indicating a role in mediating *Rickettsia felis* internalization into ISE6 cells [15].

The protein encoded by Contig4707 was found to share 80.4% and 71% aa identity to a fragment of α2-macroglobulin in *A. cajennense* and *I. scapularis*, respectively. However, the identity between the aa sequence predicted from Contig4707 and α2-macroglobulin precursor splice variant 1 in *O. moubata* was only 29%. α2-macroglobulin in *O. moubata* was isolated and characterized from plasma [16], and later the α2-macroglobulin gene was cloned in the same soft tick species [17]. α2-macroglobulin was shown to be expressed in all life stages of the hard tick *I. ricinus* with the highest expression in haemolymphs, salivary glands and ovarioles, but not in midguts [18]. Further RNAi studies indicated that challenged ticks had compromised ability to phagocytize *Chryseobacterium indologenes* but not *Borrelia burgdorferi* and *Staphylococcus xylosus* [18].

Paramyosin was initially isolated from large filaments of unstriated muscles of molluscs [19] and was suggested to play a critical role in determining the length and stability of muscle filaments in nematodes [20]. The full-length of paramyosin was 872 aa based on two unigenes, Contig2051 and Contig27282. The protein was expressed in all tissues and all developmental stages of *R. microplus* but was not found in the saliva [21]. As a component of tick myofibrils, it was also shown to have antibiotic activity. Recombinant paramyosin was able to bind IgG and collagen [21]. Further studies revealed that *R. microplus* paramyosin could induce an immune response during tick infestations. *R. microplus* paramyosin also showed a high transcription rate in organs which did not have a highly-developed musculature like fat bodies. These observations suggested the presence of additional, non-muscle related functions during tick-bovine interactions [22].

Actin is a major component of muscles in animals and exists in almost all muscle and non-muscle cell structures of eukaryotes. The aa sequences of actin are highly conserved between species. The homology of its genes between human and *Drosophila* is more than 93% [23]. In ticks, *H. flava* shares a 100% homology with *R. microplus* and 99% with *O. moubata* [24].

The full-length microplusin was estimated to be 159 aa. We compared the microplusin sequences of *H. flava* with *Amblyomma maculatum*, *Amblyomma triste* and *A. cajennense*, and the homology was found to be 74%, 73% and 71%, respectively. The homology between *H. flava* and *R. microplus* microplusin was only 29%. The full-length elongation factor-2 in *H. flava* was estimated to be 842 aa, and that of mucin was 117 aa. Mucin of *H. flava* shared 81% homology with that of *H. longicornis*. The estimated full-length of a cysteine-rich protein was 117 aa and shared a homology of 42% with that of *I. scapularis*. The biological functions of the four proteins are unknown, and further studies are needed to understand their roles.

Chitinase hydrolyses are the β-1,4 glycosidic linkages of N-acetylglucosamines. Contig15192 encoded putative chitinase in *H. flava* and has a full-length ORF of 494 bp encoding a peptide with 128 aa. Its aa identity with that in *A. cajennense* was 73.8%. Immunization of rabbits with recombinant chitinase reduced feeding efficiency and prevented moulting in *H. longicornis* [25, 26]. A recent study revealed that silencing chitinase in *A. americanum* harmed the tick cement cone stability [27], indicating that chitinase could be used as a novel acaricide. It is notable that the chitinase identified in the present study had low aa identity with that in *H. longicornis* and *A. americanum*, suggesting that it could be a different protein. Thus, the function of chitinase in *H. flava* needs further clarification.

## Conclusions

In total, 21 proteins were identified in the faecal proteome of *H. flava* females with high confidence. Together with previous *H. flava* salivary gland and midgut transcriptomes, it was demonstrated that 18 proteins were from ticks as their corresponding genes could be found in those datasets. Thus, the proteomics informed by transcriptomics (PIT) in the present study is a feasible tool to identify proteins. This tool will further facilitate studies on the biology of blood meal digestion and provide clues for the control of tick infestations. Moreover, the present study also highlights the complexity of faecal protein components, which mirrors the complexity of the process of blood digestion in ticks. More investigations are needed to elucidate the roles of these proteins in blood meal processing, interactions between ticks and hosts, and interventions in tick-borne pathogen transmission.

## Abbreviations

aa: Amino acids
ACN: Acetonitrile
APTT: Activated partial thromboplastin time
BCA: Bicinchoninic acid
DTT: 1,4-dithiothreitol
HPLC: High performance liquid chromatography
IAA: Iodoacetamide
LC/MS-MS: Liquid chromatography-tandem mass spectrometry
NCBInr: National center for biotechnology information non-redundant
NSI: Nanospray ionization
PIT: Proteomics informed by transcriptomics
PT: Prothrombin time
RACE: Rapid-amplification of cDNA ends
SDS-PAGE: Sodium dodecyl sulfate polyacrylamide gel electrophoresis
TT: Thrombin time
